# Diagnostic Accuracy of MMP-8 and IL-6-Based Point-of-Care Testing to Detect Peritoneal Dialysis-Related Peritonitis: A Single-Center Experience

**DOI:** 10.3390/diagnostics14111113

**Published:** 2024-05-27

**Authors:** Rania Ibrahim, Mido Max Hijazi, Fadwa AlAli, Abdullah Hamad, Ahlam Bushra, Lutz Mirow, Timo Siepmann

**Affiliations:** 1 Department of Nephrology, Dialysis Division, Fahad Bin Jassim Kidney Center, Hamad General Hospital, Hamad Medical Corporation, Doha P.O. Box 3050, Qatar; ribrahim4@hamad.qa (R.I.); falali1@hamad.qa (F.A.); ahamad9@hamad.qa (A.H.); aali2@hamad.qa (A.B.); 2 Division of Health Care Sciences, Dresden International University, Freiberger Str. 37, 01067 Dresden, Germany; 3 Department of Neurosurgery, Division of Spine Surgery, Faculty of Medicine, University Hospital Carl Gustav Carus, Technische Universität Dresden, Fetscherstrasse 74, 01307 Dresden, Germany; mido.hijazi@ukdd.de; 4 Department of Surgery, Klinikum Chemnitz gGmbH, Medical Faculty and University Hospital Carl Gustav Carus, Medical Campus Chemnitz, Technische Universität Dresden, Flemmingstraße 2, 09116 Chemnitz, Germany; l.mirow@skc.de; 5 Department of Neurology, Technische Universität Dresden, Medical Faculty and University Hospital Carl Gustav Carus, Fetscherstrasse 74, 01307 Dresden, Germany

**Keywords:** end-stage renal disease, peritoneal dialysis, peritoneal dialysis-related peritonitis, point-of-care testing, biomarkers, matrix metalloproteinase-8, diagnostic accuracy

## Abstract

Background: Peritoneal dialysis-related peritonitis (PDRP) is the most common complication of peritoneal dialysis (PD), which can lead to poor outcomes if not diagnosed and treated early. We aimed to investigate the diagnostic accuracy of MMP-8 and IL-6-based point-of-care tests (POCTs) in diagnosing PDRP in PD patients. Methods: This retrospective chart review study was conducted at a comprehensive kidney center in Qatar. It involved all adult PD patients who underwent PDRP from July 2018 to October 2019 and for whom MMP-8 and IL-6-based POCTs were used to diagnose presumptive peritonitis. Measures of diagnostic accuracy were computed. Peritoneal fluid effluent analysis was the reference standard. Results: We included 120 patients (68 [56.7%] females, ages 55.6 ± 15.6 years, treatment duration 39.5 ± 30.4 months [range: 5–142 months]). In this population, MMP-8 and IL-6-based POCTs yielded 100% in all dimensions of diagnostic accuracy (sensitivity, specificity, positive and negative predictive values). Conclusions: MMP-8 and IL-6-based POCTs might be helpful in the early detection of PDRP. This monocentric observation requires further confirmation in a prospective multicentric setting.

## 1. Introduction

Peritoneal dialysis (PD) is an alternative home-based dialysis option that offers many advantages for end-stage kidney disease (ESKD) patients: relatively low cost, greater autonomy, and independence, especially for the elderly with physical, cognitive, or psychological barriers [1,2]. This approach improves patients’ quality of life and reduces the financial burden on the healthcare system [3]. In Qatar, the prevalence of ESKD patients undergoing renal replacement therapy (RRT) was 666 per million people [4]. Over 15% of these patients depend on PD [5]. This utilization rate of PD in Qatar exceeds that of the USA (6.9%), Europe (5%), and the KSA (5.1%) [6]. That raises the importance of a comprehensive protocol and advanced techniques to enhance the delivery of an adequate dialysis dose to a more significant percentage of patients and minimize peritoneal membrane damage. The nature of peritoneal dialysis of the repeated infusion of dialysis fluid in the peritoneal cavity, which contains high concentrations of glucose or other osmotic agents, triggers cellular and molecular processes, leading to inflammation and fibrosis of the peritoneal membrane. Significantly, peritonitis episodes enhance the inflammatory status and accelerate the progression of peritoneal injury [7]. The reported survival of the PD modality is approximately 50% in the first 3 to 5 years, with fewer than 4% of patients maintaining PD for over 7 years [8]. That raises the importance of the early detection and confirmative diagnosis of peritoneal dialysis-related peritonitis (PDRP), which is crucial, as any delay in initiating treatment is associated with elevated morbidity and mortality [8,9,10].

PDRP remains the most serious complication of PD; it can lead to peritoneal membrane ultrafiltration failure, which can trigger shifting patients to hemodialysis (HD) and is accompanied by various complications and mortality [9,11,12]. It also increases the burden on healthcare facilities [13]. Multiple risk factors can enhance the incidence of peritonitis and related complications among PD patients, including age, female sex, body mass index (BMI), lower socioeconomic status, coronary artery disease, chronic lung disease, hypertension, and smoking [14,15,16,17]. The prognosis of PDRP relies on various factors, including patient recognition of peritonitis symptoms, immediate attendance to a health facility, early detection, immediate intervention, and initiation of empirical antibiotics; all those factors are essential to enhance the treatment of inflammation and abdominal pain and protect the integrity of the peritoneal membrane [10,17,18], which decreases peritonitis complications, prolonged hospitalization, and reduces healthcare burden. Late diagnosis of peritonitis directly influences patient survival of peritoneal dialysis [19,20]. The early recognition of signs and symptoms associated with PDRP plays a crucial role in initiating the PDRP diagnostic protocol. Therefore, in cases where PDRP is suspected, patients must inform their healthcare provider for urgent evaluation, precise diagnosis, and the appropriate therapeutic measures [16,21]. The early administration of empirical antibiotics is vital for the faster resolution of the inflammation process, decreasing abdominal pain, protecting peritoneal membrane integrity, and improving patient survival [11,12]. The existing approach for diagnosing PDRP is time-consuming: involving the cell count in peritoneal fluid and waiting for culture confirmation, which takes several hours or more and might delay the prompt administration of antibiotics, potentially impacting patient outcomes [10]. An effective and accurate test for identifying PDRP is vital for quicker diagnosis and helps avoid the typical delays in antibiotic treatment seen with conventional standard care approaches.

Enhancing patient outcomes could be achieved by a rapid point-of-care (POC) test that rapidly confirms or rules out the diagnosis of PDRP, preventing delays in initiating antibiotic treatment [17]. According to the International Society of Peritoneal Dialysis (ISPD), two or more criteria are needed to confirm PDRP in peritoneal dialysis patients, which is considered the gold standard of PDRP: 1. The clinical features are (abdominal pain and/or cloudy dialysis effluent). 2. The dialysis effluent with a white cell count exceeding 100/mL (or greater than 0.1 × 10^9^/L after a dwell time of at least two hours), with over 50 percent of cells being polymorphonuclear cells (PMN) [9]. Various commercial test strips have been explored for the early detection of PDRP, quickly and effectively at the patient’s bedside, with a high negative *p*-value of over 98%, and offer a faster turnaround time than the standard culture method [22,23,24].

Fahad Bin Jassim Center is the main dialysis center in Qatar, with a rapid increase in patient numbers [5,25]. Continuous ambulatory peritoneal dialysis (CAPD) remained the original PD mode, with significant numbers of patients being switched to automated peritoneal dialysis (APD) over the years. PD patients are trained to be alert for the signs and symptoms of PDRP and are advised to notify their dialysis provider for urgent evaluation once they suspect PDRP [9]. According to our latest quality improvement report, the estimated rate of PDRP is 0.27 episodes/patient year in 2023, compared to Europe, which has an estimated rate of 0.303 episodes/patient year in 2019 [10], while, according to the latest ISPD recommendation in 2022, the overall peritonitis rate should not exceed 0.40 episodes per year at risk. The target percentage of patients free from peritonitis per unit at any time should be more than 80% per year [9]. Many preventive measures are designed to lower the occurrence of PDRP, including administering prophylactic antibiotics immediately before catheter insertion, selecting the PD catheter placement technique, and maintaining the integrity of the exit site for seven days following PD catheter placement [26]. No evidence demonstrates the superiority of any specific catheter placement technique over another in preventing catheter-related infections [27]. Despite implementing PDRP preventive measures, including adherence to ISPD guidelines, technical improvements, comprehensive patient training, early symptom recognition, and facilitated communication with our PD center, PDRP remains a prevalent complication in our facility. To address this challenge, we implemented an efficient bedside test for early PDRP detection, leading us to explore the potential of a novel point-of-care test (POCT) (PERiPLEX^®^). This POCT was introduced to our practice between July 2018 and October 2019. The test detects two recognized markers of infection, Matrix MetalloProteinase-8 (MMP-8) and Interleukin-6 (IL-6), in a lateral flow assay using peritoneal dialysate taken from the dialysis effluent [28,29].

Consequently, we aimed to evaluate the sensitivity and efficacy of MMP-8 and IL-6-based POCT for the early detection of PDRP; we conducted a retrospective study to avoid delays in initiating empirical antibiotics therapy. We performed a retrospective study to evaluate the efficacy of the point of care to detect PDRP. In this study, we hypothesize that MMP-8 and IL-6-based point-of-care testing will perform comparably to the gold standard test for PDRP in peritoneal dialysis patients. It will demonstrate high diagnostic accuracy and potential as a reliable alternative for the early detection of PDRP. 

## 2. Materials and Methods

### 2.1. Study Design

This retrospective observational data review includes 284 PD patient records between June 2018 and October 2019 in Fahad Bin Jassim Kidney Center, the largest dialysis center in Qatar. The center serves around 300 PD patients and has a particular area for the training and aftercare of patients with CAPD and APD [4,5,25,30].

**Figure 1:**
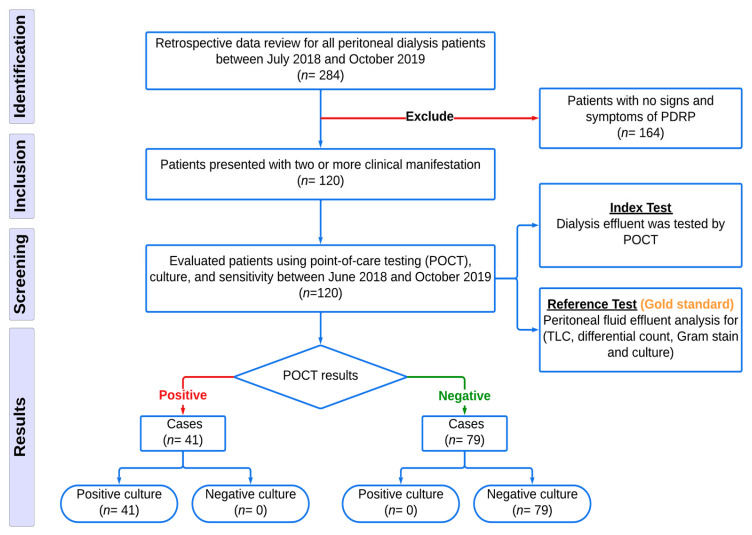
Flow diagram for PD patients’ identification and screening for PDRP in Qatar between (July 2018 and October 2019). (**PDRP**) peritoneal dialysis-related peritonitis; (**TLC**) total leukocyte count. **Index test**: This evaluates MMP-8 and IL-6-based POCT for its accuracy, sensitivity, and specificity to detect or roll out peritoneal dialysis-related peritonitis. **Reference test**: This is the **gold standard** for diagnosing peritoneal dialysis-related peritonitis. According to the International Society of Peritoneal Dialysis (ISDP) guidelines, it is considered the most accurate and reliable diagnostic test.

We investigated 120 patients who were presented with two or more signs and symptoms of PDRP and tested them using MMP-8 and IL-6-based POCT to detect PDRP in Fahad Bin Jassim Kidney Center between June 2018 and October 2019 (Figure 1).

The figure shows the screening of 284 patients performing home peritoneal dialysis—120 patients presented with PDRP signs and symptoms (Figure 1).

### 2.2. Study Objectives

We aimed to acquire pilot data on the diagnostic accuracy of POCT in diagnosing PDRP in peritoneal dialysis patients.

### 2.3. Institutional Review Board and Electronic Patient Data Software

This study was reviewed and approved by the Institutional Reviewer Board for the Medical Research Center at Hamad Medical Corporation Qatar MRC-01-23-781. The IRB waived the informed consent, giving the study a retrospective design. Data were identified using the electronic health record at HMC (CERNER Millennium is an electronic health record (EHR) system used in Hamad Medical Corporation to manage patient information and clinical workflows.).

### 2.4. Study Population

Based on the medical records review, 120 PD patients with two or more clinical manifestations of PDRP were presented to our dialysis center and tested using POCT to detect the presence of PDRP between June 2018 and October 2019.

#### 2.4.1. Inclusion Criteria

We screened all adult ambulatory PD patients 18 years or older, routinely followed at Fahad Bin Jassim Kidney Center, and tested by POCT for suspected PDRP. 

#### 2.4.2. Exclusion Criteria

All pediatric patients less than 18 years old were excluded.

### 2.5. Case Definitions

**Figure 2:**
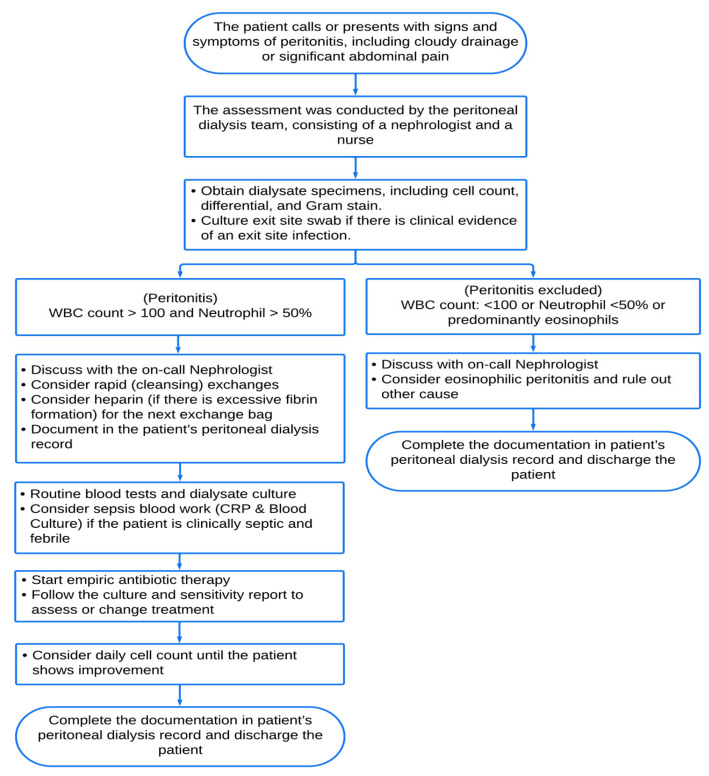
The clinical pathway outlines the diagnostic protocol for suspected patients with peritoneal dialysis-related peritonitis (PDRP). **WBC**: White blood cells, **CRP**: C-reactive protein.

**Figure 3:**
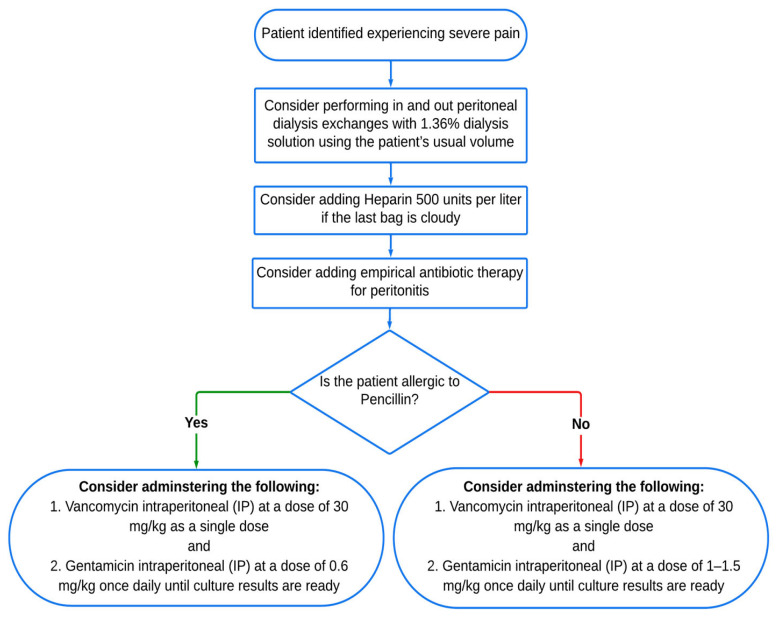
The empirical antibiotic treatment protocol against Gram-positive and Gram-negative microorganisms for suspected patients with PDRP. **IP**: Intraperitoneal Cavity.

Our diagnostic approach for PDRP (Figure 2) is based on the ISPD guidelines. Diagnosis requires meeting at least two of the following three criteria: (a) presence of clinical features (abdominal pain and/or cloudy dialysis effluent); (b) peritoneal fluid effluent total leukocyte count (TLC) of >100 WBC/mm^3^ with >50% polymorphonuclear (PMN) leukocytes; (c) positive culture from dialysis effluent [9,31,32]. These diagnostic criteria are considered the gold standard in PDRP practice. For all suspected PDRP cases, the unit protocol was followed by sending the peritoneal fluids for aerobic, anaerobic, and fungal cultures, along with cell count and differential analysis: (a) 5 mL of dialysate effluent in an EDTA tube sent to the hematology laboratory for cell count. (b) An amount of 100 mL of dialysate effluent in a sterile specimen bottle for microbiological analysis (including Gram staining and Cytospin concentrate inoculation in blood culture bottles conducted in the microbiology laboratory). (c) Empirical antibiotic administration was initiated after obtaining the appropriate peritoneal samples and performing the clinical examination (Figure 3). If the patient has no fluid in the peritoneal cavity on arrival, instill 1 L of dialysate (or as per prescriber) volume of dialysate and let it dwell for a minimum of 2 h before collecting samples. The penicillin allergy status was determined. In case of adverse penicillin allergy, vancomycin intraperitoneal (IP) 30 mg/kg administered for one dose and ceftazidime intraperitoneal (IP) 1–1.5 g administered once daily until the culture result was ready, and if the allergic test was positive, vancomycin intraperitoneal (IP) 30 mg/kg one dose and gentamicin intraperitoneal (IP) 0.6 mg/kg administered once daily until culture result was ready. The dwell time in all cases was 6–8 h—the maximum vancomycin dose was 2.5 gm (IP). The vancomycin dose was 30 mg/kg (should be rounded to 500 mg). The vancomycin level by day 3–5 and re-dosed if below 15 mcg/mL was considered; no evidence that monitoring aminoglycoside levels mitigates toxicity risk or enhances efficacy. According to our unit protocol, gentamicin or cefepime may be used instead of ceftazidime. The minimum antibiotic therapy is two weeks; three weeks was recommended for more severe infections. The length of treatment was determined by clinical response and the type of pathogen; the clinical improvement was inspected within 72 h of initiation of the antibiotic therapy, and if there was no improvement in peritonitis, re-culture in 96 h (about 4 days), and a change in antibiotics was considered. Multiple PDRP was defined as relapsing, recurrent, repeated PDRP occurring after four weeks of treatment with a different organism.

### 2.6. Data Collection

All cases were identified from Qatar’s national electronic medical records (CERNER) to retrieve the following data: demographic and clinical characteristics, comorbidities at the start of dialysis, including coronary artery disease, peripheral vascular disease, cerebrovascular disease, chronic lung disease, diabetes, and hypertension, results of the peritoneal fluid effluent analysis (TLC, differential count, Gram stain, and culture), and the PDRP outcomes.

### 2.7. Study Procedure and Interpretation

**Figure 4:**
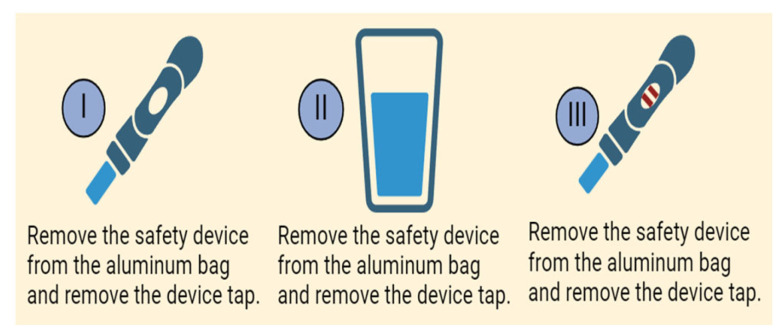
The application of MMP-8 and IL-6-based POCT at bedside (Artwork by R. Ibrahim). Created with BioRender.com.

**Figure 5:**
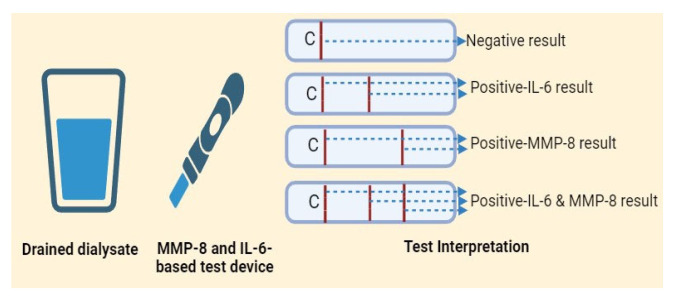
Interpretation of the test results displayed in the device window (Artwork by R. Ibrahim). Created with BioRender.com.

The PD nurses conducted POCT for all patients showing signs or symptoms of PDRP; a sample of drained effluent fluid was tested by Perplex at the bedside (after a dwell time of at least two hours) (Figure 4). According to the manufacturer’s recommendations, the test is considered positive for PDRP if one or both test lines appear in the test strip window. Additionally, another sample of effluent fluid was forwarded to the lab for TLC, differential count, culture, and sensitivity. The empirical antibiotic was administered immediately based on the POCT results without waiting for the laboratory results. The test is considered positive for PDRP if one or both test lines appear in the test strip window (Figure 5).

### 2.8. Statistical Analysis

The results were expressed as frequencies and percentages for categorical variables and mean ± standard deviation (SD) for continuous variables. In all cases, the results of the POCT were compared to the PDRP gold standard test [33]. The equation to calculate efficacy measures is as follows:

- Sensitivity = number of patients with true positive test/number of patients with true positive test + number of patients with false negative test.
- Specificity = number of patients with true negative test/number of patients with true negative test + number of patients with false positive test.
- Positive predictive value = number of patients with true positive test/number of patients with true positive test + number of patients with false positive test.
- Negative predictive value = number of patients with true negative test/number of patients with true negative test + number of patients with false negative test.

The accuracy of the POCT was expressed as sensitivity, specificity, negative predictive value (NPP), and positive predictive value (PPV). Data analysis was performed with SPSS software (IBM SPSS Statistics for Windows, Version 25.0. IBM Corp: Armonk, NY, USA).

## 3. Results

### 3.1. Baseline Characteristics

During the study period, we reviewed the medical records of 120 patients, 52 (43.3%) males and 68 (56.7%) females. The mean age was 55.6 ± 15.6 years (range: 23–90 years), and 25 (20.8%) patients were of Arab nationality. The peritoneal dialysis treatment mean duration was 38.5 ± 30.4 months (range: 5–142 months). Hypertension and diabetes mellitus were found in 91.7% and 47.1% of the cases, respectively. The most frequent presenting symptom was abdominal pain with 67 (57.8%), followed by fever with 33 (28.4%). The patient’s demographic and clinical characteristics are described in (Table 1 and Table 2).

### 3.2. POCT and Other Test Results in Comparison with the Reference Standard

PDRP was confirmed in 41 (34.2%) cases by both the gold standard test and POCT. The sensitivity, specificity, PPV, and NPV of the POCT were 100%, 100%, 100%, and 100%, respectively, with 100% accuracy. The accuracy tests of the POCT, clinical presentation, TLC > 100/mL or PMN > 50%, and culture are described in (Table 3 and Table 4).

## 4. Discussion

Our study aim was to evaluate the efficacy and the diagnostic accuracy of the MMP-8 and IL-6-based point-of-care testing versus the gold standard test for diagnosing PDRP in peritoneal dialysis patients. It unveiled several key findings. The MMP-8 and IL-6-based point-of-care testing demonstrated excellent diagnostic precision, elevated sensitivity, specificity, and predictive value levels and provided results statistically similar to the gold standard PDRP test.

Our analysis of a cohort of patients undergoing PD in Qatar supports the usefulness of POCT for the early detection of PDRP, displaying outstanding performance in all measures of diagnostic accuracy. 

Most previous studies on POCT to detect PDRP used urinary reactive strips, which rely on identifying esterase activity in granulocyte leukocytes to evaluate leukocyte concentration and detect nitrites produced by nitrate activity from specific bacteria. Test accuracy varied among studies, with sensitivity between 80% and 100% and specificity ranging from 45% to 95% [23,34,35,36]. Other new biomarkers and technologies have also been developed to detect early PDRP, for instance, Mark Buckup used the prototype technology of microscopy and image analysis as a screening tool for peritonitis to improve the overall PD experience. Also, Guiling Liu’s nomogram model identifies patients at high risk of Gram-negative bacterial (GNB) infections in patients with peritoneal dialysis-related peritonitis, which contributes to timely intervention to improve patient prognosis [29,37,38,39]. 

PDRP lies at the top of the hierarchy of importance of PD outcomes [40]. The prevalence of PDRP in Qatar has consistently aligned with the ISPD target throughout the years [31,41]. Unfortunately, PDRP cases have detrimental effects on peritoneal membrane function, hospitalization, temporary or permanent transition to HD, and PD-related mortality [9,42,43]. 

We analyzed the type of pathogen in Table 2. As expected, Gram-positive cocci were the most common, while fungal was the least common. As our POCT detects inflammatory markers, it was able to detect APAP regarding the type of pathogens. Despite having a wide range of pathogen etiology for PDRP (Table 2), there was no significant correlation with our POCT test result as the test was 100% sensitive and specific. Administering antibiotic treatment is crucial for improving outcomes in cases of PDRP. Previous research showed there is a 6.8% increase in the risk of peritoneal dialysis failure or death for each hour of delay in initiating antibacterial therapy from the moment of hospital presentation [17]. The association between the timing of antibiotic initiation and the prognosis of PDRP is largely unknown [17]. In our cohort of PD patients, antibiotics were routinely administered after receiving cell count results, typically taking 5–8 h on average. However, empirical antibiotics were initiated immediately upon a positive test result, resulting in a substantial timesaving of approximately 8 h to control the infection. The distribution of PDRP severity was as follows: mild (50%), moderate (13%), and severe (37%). Of this population, 21.7% required admission to the hospital for further interventions. Our internal data showed that the average hospitalization of patients presenting with PDRP in our center is 30–40%, which shows a potential benefit in reducing hospitalization with the utilization of POCT. Implementing POCT for the early detection of PDRP might positively influence the prognosis and mitigate the adverse consequences of this PDRP.

Our study provided the early detection of 41 cases of PDRP, using MMP-8 and IL-6-based POCT after presenting with a clinical suspicion of PDRP abdominal pain and/or cloudy dialysis effluent), and confirmed by a gold standard test [32]. The ideal POCT should demonstrate superior diagnostic accuracy with elevated sensitivity, specificity, and predictive values. It should also be a simple and rapid process to minimize delays and improve cost-effectiveness [29].

Our study showed higher diagnostic accuracy for the MMP-8 and IL-6-based POCT compared to previously evaluated urinary strips [34,44]. The PERiPLEX^®^ system utilizes a lateral flow assay to MMP-8 and IL-6, utilizing peritoneal dialysate derived from a dialysis effluent as significant inflammatory indicators with high concentrations during sepsis cases [29,45]. MMP-8 is among the top 5 biomarkers for distinguishing bacterial peritonitis from sterile peritoneal inflammation or viral infection [46]. Consequently, MMP-8 and IL-6-based POCT might be superior to urinary strips in detecting PDRP, rapidly diagnosing or excluding PDRP, and preventing delays in initiating antibiotic therapy [17]. An evaluation of PERiPLEX^®^ yielded identical values for sensitivity and a negative predictive value but lower specificity and positive predictive value (88%) [44]. Taken together, our analysis, viewed in conjunction with previous investigations, suggests that the PERiPLEX^®^ POCT might be beneficial in the early detection of PDRP.

### Limitations and Strengths of this Study

This retrospective study is monocentric, limiting its generalizability. Our study’s strengths comprise the high consistency and standardization in implementing the POCT in clinical practice, ensuring high internal validity. It has to be noted that, per design, we were unable to evaluate the time between the onset of symptoms and performing the POCT, as well as the time between the onset of symptoms and the receipt of TLC with the differential laboratory results.

However, our diagnostic accuracy analysis forms substantiate a basis for a prospective multicenter study to further explore and confirm our observation of the usefulness of MMP-8 and IL-6-based POCT in the early detection of PDRP. Moreover, our data might be useful for calculating the required sample size to confirm diagnostic accuracy prospectively. This might also be helpful to the design of randomized controlled interventional studies on the treatment of PDRP [47].

## 5. Conclusions

In conclusion, this study aimed to evaluate the efficacy of MMP-8 and IL-6-based POCT compared with the gold standard test in detecting PDRP. MMP-8 and IL-6-based POCT had high sensitivity, specificity, and positive and negative predictive values. It might also be helpful in the early detection of PDRP. This monocentric retrospective observation has limited external validity per the study design but provides a basis for further exploration in a prospective multicentric setting.

## Figures and Tables

**Table 1:** Demographic and clinical characteristics of the patients involved in the study.

Characteristics	*N* (%)/(Mean ± SD)
Age (Mean ± SD)	55.6 ± 15.6 (range: 23–90 years)
Gender	
Male	52 (43.3)
Female	68 (56.7)
Nationality	
Arab national (Qataris)	25 (20.8)
Arab non-national (Arab non-Qataris)	44 (36.7)
South Asian (Pakistani/Indian)	30 (25)
Other mixed nationalities	21 (17.4)
Comorbidities Diabetes mellitus	56 (47.1)
Diabetic nephropathy	48 (40.3)
Eye disease	
Retinopathy	15 (12.5)
Blindness	2 (1.7)
Others (Glaucoma, Cataracts)	13 (10.8)
Neuropathy	15 (12.6)
Hypertension	110 (91.7)
Pulmonary disease	
Asthma	8 (6.7)
Interstitial lung disease	2 (1.7)
Others (IPF, COPD)	9 (7.6)
Dyslipidemia	46 (38.7)
Hypoparathyroidism (post-surgical)	2 (1.7)
Hyperparathyroidism (secondary to renal failure)	16 (13.4)
Hypothyroidism	16 (13.4)
PD duration (Mean ± SD)	38.5 ± 30.4 (range: 5–142 months)
Hyperthyroidism	0 (0)
Oncology disease	
Breast cancer	1 (0.8)
Gynecological malignancy	1 (0.8)
Others (leukemia, lung cancer)	5 (4.2)
Peripheral vascular disease	
Diabetic foot	3 (2.5)
Others (Gangrene, Amputation)	2 (1.7)
Cardiac disease	
Coronary artery disease	25 (20.8)
Angina	3 (2.5)
Cardiomyopathy	4 (3.4)
Heart failure	12 (10.1)
Arrhythmia	3 (2.5)
Valvular heart disease	3 (2.5)
Others (Angina, MI)	13 (10.9)
Neurological diseases	
Ischemic stroke	14 (11.8)
Intracranial/hematoma	3 (2.5)
Epilepsy	5 (4.2)
Others (MS, CVA, TIA)	1 (0.8)

**IPF**: idiopathic pulmonary fibrosis; **COPD**: chronic obstructive pulmonary disease; **MI**: myocardial infarction, cardiomyopathy; **MS**: multiple sclerosis; **CVA**: cerebrovascular accident, **TIA**: transient ischemic attack.

**Table 2:** Clinical characteristics of the patients involved in this study.

Clinical Characteristics	*N* (%)
Clinical presentation	
Abdominal pain	67 (57.8)
Fever	33 (28.4)
Vomiting	14 (12.1)
Diarrhea	5 (4.3)
Cloudy outflow	19 (16.4)
Hazy outflow	10 (8.6)
Low BP	7 (5.8)
Peritoneal fluid effluent tests	
POCT results	41 (34.2)
Culture results	40 (33.3)
WBC (TLC)	
Less than 100 (No PDRP)	78 (65.0)
Between 100 to 1000 (Mild)	21 (17.5)
Between 1000 to 3000 (Moderate)	6 (5.0)
More than 3000 (Severe)	15 (12.6)
Neutrophil (PMN) (>50%)	41 (34.2)
Confirmed PDRP	41 (34.2)
Type of organisms	
Gram-positive	21 (52.5)
Gram-negative	12 (30)
TB	1 (2.5)
Fungal	1 (2.5)
No growth	5 (12.5)
Treatment	40 (33.3)
Admission	26 (21.7)

**PD**: peritoneal dialysis; **PDRP**: peritoneal dialysis-related peritonitis; **POCT**: point-of-care testing; **BP**: blood pressure; **WBC**: white blood count; **TLC**: total leukocyte count; **PMN**: polymorphonuclear; **TB**: tuberculosis.

**Table 3:** The results of POCT for diagnosis of PDRP.

	Clinical Evaluation (*n* = 41)	TLC > 100/mL and PMN > 50% (*n* = 41)	Culture (*n* = 40)	MMP-8/IL-POCT Positive (*n* = 41)
PDRP (*n* = 41)	41	41	40	41
No PDRP (*n* = 79)	0	0	0	0
Sensitivity	100%	100%	97.56%	100%
Specificity	100%	100%	100%	100%
PPV	100%	100%	100%	100%
NPV	100%	100%	98.75%	100%
Accuracy	100%	100%	99.16%	100%

**PDRP**: peritoneal dialysis-related peritonitis; **PPV**: positive predictive value; **NPV**: negative predictive value.

**Table 4:** Diagnostic accuracy of different variables to detect PDRP.

		PDRP		Total
		Yes	No	
MMP-8 and IL-6-based POCT test results	Positive	41	0	41
	Negative	0	79	79
Total		41	79	120

**PDRP**: peritoneal dialysis-related peritonitis.

## Data Availability

The original contributions presented in the study are included in the article; further inquiries can be directed to the corresponding author/s.
